# Sphingolipids in Type 1 Diabetes: Focus on Beta-Cells

**DOI:** 10.3390/cells9081835

**Published:** 2020-08-04

**Authors:** Ewa Gurgul-Convey

**Affiliations:** Institute of Clinical Biochemistry, Hannover Medical School, Carl-Neuberg-Str.1, 30625 Hannover, Germany; Gurgul-Convey.Ewa@mh-hannover.de

**Keywords:** type 1 diabetes, beta-cells, islets, insulin, cytokines, sphingolipids, S1P, animal models

## Abstract

Type 1 diabetes (T1DM) is a chronic autoimmune disease, with a strong genetic background, leading to a gradual loss of pancreatic beta-cells, which secrete insulin and control glucose homeostasis. Patients with T1DM require life-long substitution with insulin and are at high risk for development of severe secondary complications. The incidence of T1DM has been continuously growing in the last decades, indicating an important contribution of environmental factors. Accumulating data indicates that sphingolipids may be crucially involved in T1DM development. The serum lipidome of T1DM patients is characterized by significantly altered sphingolipid composition compared to nondiabetic, healthy probands. Recently, several polymorphisms in the genes encoding the enzymatic machinery for sphingolipid production have been identified in T1DM individuals. Evidence gained from studies in rodent islets and beta-cells exposed to cytokines indicates dysregulation of the sphingolipid biosynthetic pathway and impaired function of several sphingolipids. Moreover, a number of glycosphingolipids have been suggested to act as beta-cell autoantigens. Studies in animal models of autoimmune diabetes, such as the Non Obese Diabetic (NOD) mouse and the LEW.1AR1-iddm (IDDM) rat, indicate a crucial role of sphingolipids in immune cell trafficking, islet infiltration and diabetes development. In this review, the up-to-date status on the findings about sphingolipids in T1DM will be provided, the under-investigated research areas will be identified and perspectives for future studies will be given.

## 1. Introduction

Sphingolipids (SLs) are a diverse family of lipid molecules playing a pivotal role in a number of autoimmune and inflammatory disorders [1,2,3,4]. The role of SLs in glucose homeostasis and insulin sensitivity is relatively well described in the context of metabolic-syndrome related type 2 diabetes (T2DM) [4,5,6,7,8,9,10,11,12]. In contrast, the importance of SLs in the beta-cell demise during autoimmune type 1 diabetes (T1DM) development has been so far less frequently addressed. Interestingly, a number of new investigations suggest that dietary fats and lipid metabolism may be considered as triggers that could induce or sensitize the autoimmunity onset in T1DM [13]. Polymorphisms in several genes encoding proteins involved in the SL pathway were recently linked to T1DM overt [14]. Moreover, profound changes in SL serum profiles upon autoimmunity development were detected in T1DM patients [14,15,16,17,18,19,20]. The last years have revealed that the enzymatic machinery and the system of receptors and transporters for bioactive SLs are significantly affected in pancreatic beta-cells by proinflammatory cytokines that are released from immune cells infiltrating islets [21]. SLs may be useful biomarkers for T1DM development [17]. In vitro studies of cytokine toxicity using genetically modified beta-cells, naturally occurring SLs and their analogues suggest that alterations of beta-cell SLs may affect insulin secretory capacity and beta-cell fate during T1DM development.

In this review various aspects of sphingolipid action and effects of the major proinflammatory cytokines on the SL pathway in pancreatic beta-cells will be discussed. Next, the engagement of SLs in the autoimmune reaction against beta-cells during T1DM development will be addressed. The present status of SL studies in animal models of autoimmune diabetes and an update on findings in T1DM patients will be summarized. Finally, perspectives, which should drive future research in the context of SLs and T1DM, will be presented.

## 2. Overview of Mechanisms of Beta-Cell Destruction in T1DM

**Figure 1:**
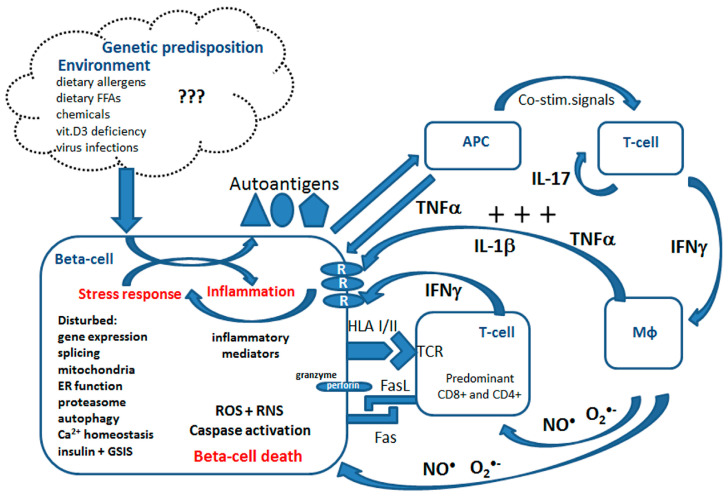
Model of cytokine-mediated beta-cell death in T1DM. In genetically predisposed individuals various environmental factors trigger the autoimmune response aimed at pancreatic beta-cells. Environmental triggers lead to beta-cell stress and release of autoantigens, which are processed and presented by antigen-presenting cells (APC). This leads to T-cell and macrophage (MΦ) activation. Consequently proinflammatory cytokines and radicals (NO^●^, nitric oxide and O_2_^●−^, superoxide anion radicals) as well as perforin-granzyme mediators are released in the vicinity of beta-cells. Proinflammatory cytokines potentiate autoimmune reaction by stimulation of CD8+ and CD4+ T-cells. Activated immune cells interact with beta-cells via FasL-Fas and also via HLAI/II-TCR systems. The action of proinflammatory cytokines requires the binding and activation of cytokine receptors (R) on beta-cells. This accelerates the multifaceted stress response and induces inflammation in beta-cells. The aggravation of the autoimmunity is achieved by biosynthesis and release of inflammatory mediators from beta-cells. Beta-cells are particularly vulnerable to the stress response and inflammation due to their weak antioxidative and anti-inflammatory defense status. Cytokines induce reactive oxygen species (ROS) and reactive nitrogen species (RNS) formation in beta-cells. Both defects in the immune response and vulnerability of beta-cells participate in the execution of beta-cell demise during T1DM development (more details in text).

Type 1 diabetes mellitus (T1DM) is an autoimmune disease with a strong genetic background, affecting millions of people worldwide, mostly in their childhood or early adolescence [22,23]. The incidence of T1DM has been significantly increasing in the last decades, similarly to other autoimmune diseases, indicating an important role of environmental factors [22,24]. During T1DM development pancreatic beta-cells are gradually destroyed due to an autoimmune reaction [22,25,26,27,28,29]. Beta-cells produce and supply our body with insulin, the most important anabolic hormone controlling blood glucose levels. The factors triggering the activation of immune cells, T-cells and macrophages, in T1DM remain unclear. It is speculated that certain food components (such as cow milk proteins or gluten), vitamin D3 deficiency, viral infections (e.g., enterovirus) and most recently saturated fats may trigger this response [13,22,26,30,31] (summarized in Figure 1). T1DM patients require a life-long substitution with insulin and are prone to severe secondary complications, such as cardiovascular dysfunction, nephropathy or retinopathy [22].

Immune cell activation is accompanied by dynamic changes in the gene expression of proteins related to inflammation and secretion of inflammatory mediators [26,27,30,32,33] (Figure 1). The infiltrate consists of a mixture of CD4+ and CD8+ T-cells, B-cells, macrophages and Natural Killer (NK) cells [31]. The activated immune cells infiltrating pancreatic islets convey their beta-cell directed cytotoxic effects via cell–cell interactions and through secretion of proinflammatory mediators. So far the research has focused predominantly on the three cytokines, namely IL-1β, TNFα and IFNγ, and their role in beta-cells is meanwhile relatively well characterized [25,27,28,29,30,31,33,34]. The intracellular effects of proinflammatory cytokines in beta-cells are pleiotropic and involve the activation of several transcription factors (NFκB, AP-1, Nova and others), disturbances of mRNA splicing, changes of the expression, activity and post-translational modifications of proteins [27,30,32,35]. Moreover, in beta-cells isolated from T1DM donors the upregulated mRNA and protein expression of Class I and Class II HLA were detected [36,37]. Several molecular processes have been associated with beta-cell death in T1DM including mitochondrial and endoplasmic reticulum (ER) stress, the generation of reactive oxygen species (ROS) and nitric oxide (NO^●^), the synthesis of inflammatory mediators, activation of immunoproteasome, no-go and nonsense-mediated RNA decay, dysregulation of calcium homeostasis and altered autophagy as well as the activation of the perforin-granzyme system [21,25,30,32,38,39,40,41,42,43,44,45,46] (summarized in Figure 1).

Beta-cells are particularly vulnerable to oxidative stress due to an imbalance in the enzymatic capacity responsible for generation and detoxification of ROS [40,42,44,45]. The superoxide dismutases, which dismutate superoxide radical anions to hydrogen peroxide (H_2_O_2_), are well expressed in the cytosolic (CuZnSOD) and in the mitochondrial compartment (MnSOD). This is in contrast to the enzymes catalyzing the detoxification of H_2_O_2_ to water, namely glutathione peroxidase (Gpx) and particularly catalase (Cat), which are very weakly expressed. Proinflammatory cytokines potentiate this imbalance specifically in the mitochondrial compartment through the induction of MnSOD expression and activity [40,44]. The increased generation rate of H_2_O_2_ together with the lack of an appropriate H_2_O_2_ detoxification system and a parallel increase of intracellular NO^●^ concentration are the perfect prerequisite for the formation of highly toxic hydroxyl radicals. This leads to intramitochondrial oxidative damage, caspase-9-dependent apoptosis induction, disturbed autophagy and beta-cell death [40,42]. Moreover, cytokines activate the ER stress response, leading to accumulation of unfolded/misfolded proteins, dysregulation of ER calcium homeostasis and induction of the proapoptotic transcription factor CHOP [47]. Additionally, cytokines induce alternative splicing and immunoproteasome activation in beta-cells, which may contribute to neoantigen formation, and aggravate the autoimmune reaction [47]. Several studies showed also that the vulnerability of beta-cells to proinflammatory cytokines is associated with an imbalance of the enzymatic capacity responsible for the generation of pro- and anti-inflammatory eicosanoids [39,48,49,50]. Our recent data indicate that MCPIP1 (monocyte chemotactic protein-induced protein 1), a strong regulator of the inflammatory response, which acts as a specific RNase, is strongly induced by proinflammatory cytokines in clonal beta-cells and upon diabetes development in the animal model of human T1DM [43]. The transcriptomic analysis of beta-cells from T1DM individuals suggests a significantly increased expression of the gene encoding MCPIP1 [37] (GEO Bioproject PRJNA497610 HLA Class II analysis of human pancreatic beta-cells). Our recent studies revealed that MCPIP1 regulates the beta-cell response to cytokine toxicity and the fine-tuning of its expression is essential for the beta-cell fate [43]. Interestingly, our preliminary observations indicate that a number of the sphingolipid pathway enzymes might be affected by this specific RNase, a mechanism that could contribute to cytokine effects on beta-cell sphingolipidome. Finally, proinflammatory cytokines dysregulate the beta-cell function by shutting down insulin biosynthesis and by disrupting glucose-stimulated insulin secretion (GSIS) [21,41,51,52,53].

## 3. Biosynthesis of Sphingolipids

**Figure 2:**
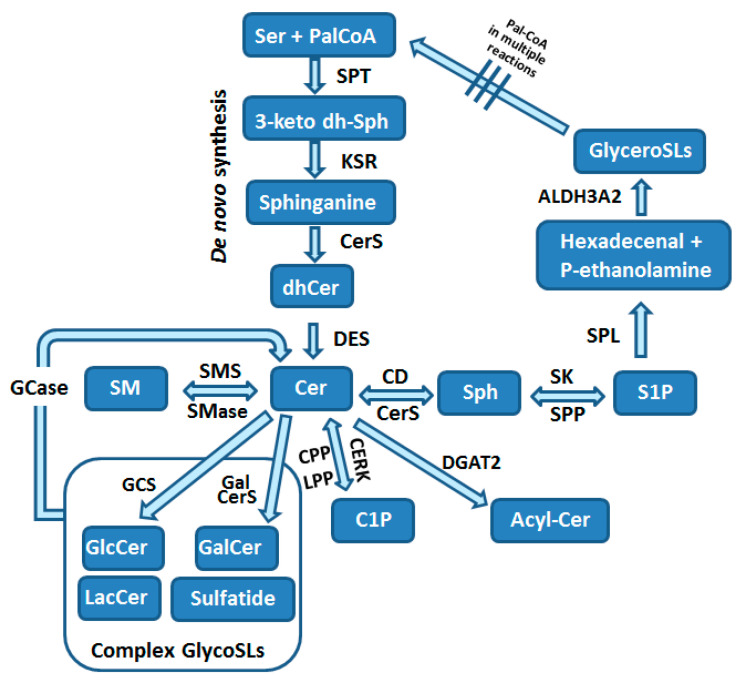
Transcriptomic data from islets and qRT-PCR results from beta-cell lines suggest that beta-cells express all genes regulating the SL pathway. The exact mRNA and protein expression level of various enzymatic components of the SL pathway in human beta-cells still needs to be characterized. Enzymes: ALDH2A3, fatty aldehyde desaturase A3, CD, ceramidase, CERK, ceramide kinase, CPP, ceramide 1-phosphate phosphatase, CerS, ceramide synthase, Des, sphingolipid-delta-4-desaturase, DGAT2, diacylglycerol acyltrasferase-2, GCS, GlcCer synthase, GalCerS, GalCer synthase, GCase, glycosidase, KSR, 3-keto sphingosine reductase, LPP, lysophospholipid phosphatase, SMS, sphingomyelin synthase; SMase, sphingomyelinase, SK, sphingosine kinase, SPP, sphingosine 1-phosphate phosphatase, SPT, serine palmitoyltransferase, SPL, sphingosine 1-phosphate lyase. Biomolecules: Cer, ceramide, C1P, ceramide 1-phosphate, dhCer, dihydroceramide, GlycoSLs, glycosphingolipids, GlyceroSLs, glycerosphingolipids, 3-keto dhSph, 3-keto dihydro sphingosine, Ser, serine, SM, sphingomyelin, Sph, sphingosine, S1P, sphingosine 1-phosphate, PalCoA, palmitoyl-coenzyme A.

Sphingolipids are composed of a polar head group and two nonpolar tails. The core of SLs is the long-chain aliphatic amino alcohol sphingosine, which is O-linked to a polar head group (e.g., ethanolamine, serine or choline) and N-linked to an acyl group of various fatty acids. SLs are the most structurally diverse lipid family, considering the hundreds of possible head groups, dozens of long-chain bases, and many fatty acids, that can be used as building blocks. The maintenance of the so-called SL rheostat is crucial for the normal function of cells and cell survival, and is compromised by a network of enzymatic reactions depicted in Figure 2. Consequently, this promotes site-specific effects and downstream targets of various SLs.

The sphingolipid pathway starts with the de novo biosynthesis of 3-ketosphinganine in the ER from L-serine and palmitoyl-CoA in the rate-limiting reaction catalyzed by serine-palmitoyltransferase (SPT; Figure 2) [3,12,54,55]. SPT can use acyl-CoAs in the range of C12 to C18, but the most typically used is the C18-CoA. Besides serine, alanine and glycine may also be used under specific circumstances, resulting in the formation of a variety of atypical sphingoid bases, which represent approximately 15% of SLs in human plasma [56]. De novo synthesis of SLs is upregulated by substrate availability and can be downregulated by mammalian orosomucoid-like (ORMDL) proteins, which form a complex with SPT and inhibit its activity [57].

In the next step of the SL biosynthesis, 3-ketosphinganine undergoes a reduction to dihydrosphingosine by 3-keto-sphinganine reductase (KSR) that is thereafter N-acylated by the action of one of six ceramide synthases (CerS1-6) to form dihydroceramide in ER [3,54]. Each of CerS types uses specific acyl chains, with saturated or monounsaturated fatty acids (C14-C26). Dihydroceramides are then dehydrogenated to ceramides by dihydroceramide desaturase (DES) [54] (Figure 2). Ceramides are the central part of the SL pathway and can be used in numerous reactions (Figure 2). Cer can be utilized for formation of (i) sphingosine, (ii) sphingomyelin, (iii) complex glycosphingolipids, (iv) ceramide 1-phosphate (C1P) or (v) acylceramide [3,54] (Figure 2).

Ceramide is deacylated by ceramidases (CDs) to form sphingosine [3,54]. This reaction can take place in different subcellular organelles and is catalyzed by distinct subtypes of CDs [3]. Sphingosine is either reutilized for Cer generation by CerS or can be phosphorylated by one of two sphingosine kinases (SK1 and SK2) to form a potent bioactive SL called sphingosine 1-phosphate (S1P; Figure 2). SK1 localizes to plasma membrane and ER, while SK2 was found in the cytosol, ER, mitochondria and nucleus [58,59,60]. This differential spatial location of two SK isoforms defines the specialized and contradictory effects of each isoforms [58,59]. Cytosolic S1P can be dephosphorylated by ER-localized S1P phosphatases (SPP1 and SPP2), while at the cell surface S1P dephosphorylation is catalyzed by lipid phosphate phosphatases (LPP1-3). These reversible reactions are crucial for SL homeostasis within the cells, and enable refilling of sphingosine into the SL pathway [5].

The final step of SL metabolism is the irreversible degradation of S1P catalyzed by S1P lyase (SPL), an enzyme localized in ER with the catalytic center facing the cytosol (Figure 2) [3,61]. S1P is degraded to hexadecenal and phosphoethanolamine, which are intermediates in the phospholipid biosynthesis pathway. The potentially toxic, when accumulated in high amounts especially within the nucleus [62,63], hexadecenal is under normal conditions effectively metabolized by the fatty acid dehydrogenase ALDH3A2 to hexadecenoate [64]. Hexadecenoate can be further utilized for palmitoyl-CoA generation within the glycerolipid pathway [64]. Additionally to ER, SPL was also found by proteomics approaches to be present in the mitochondrial associated membranes (MAMs), the membrane crossroads of mitochondria and ER, which is involved in cell signaling and metabolite exchange [65]. These observations suggest a potential participation of SPL in unique functions of mitochondria and MAMs [66].

Ceramide can be transported to other subcellular locations by vesicular transport or with help of the ceramide transfer protein CERT [3,54]. In mammalian cells CERT function is regulated by SL levels through the PKD-dependent phosphorylation [67]. In the trans-Golgi Cer can be converted to sphingomyelin by sphingomyelin synthase (Figure 2) (SMS1) [3,54]. Another isoform of SMS, namely SMS2, is localized in plasma membrane [3]. Sphingomyelin can be metabolized back to Cer by sphingomyelinases (SMase), which are found in plasma membrane, the Golgi apparatus and in mitochondria-associated membranes (Figure 2) [3,54]. In the cis-Golgi Cer is converted by glucosylceramide synthase (GCS) to glucosylceramide (GluCer; Figure 2) [68]. Galactosylceramide (GalCer) is produced by GalCer synthase (Figure 2) [68]. The formation of complex glycosphingolipids (glycoSLs) requires the transport of GluCer to trans-Golgi network by four-phosphate adaptor protein 2 (FAPP2) that also regulates trafficking of vesicles from Golgi to plasma membrane [3]. Lactosylceramide (LacCer) is then produced by LacCer synthase transferring galactose from UDP-galactose to GluCer [68]. The more complex glycoSLs all use LacCer as a common backbone. The generation of multiple glycoSL species takes place by the action of specific enzymes catalyzing the stepwise addition of sugar monomers, which branch to form complex chains [68]. Complex glycoSLs are then transported from the Golgi to their target location, which is typically the plasma membrane [68]. Sphingomyelin and glycoSLs have been shown to be transported in a vesicle-dependent manner [3,68]. Both SM and glycoSLs undergo lysosomal degradation by glycosidases and acid SMase, respectively, that are responsible for the removal of head groups to form ceramides (Figure 2) [68].

Alternatively, ceramide can be also phosphorylated to ceramide 1-phosphate (C1P), a bioactive SL, by ceramide kinase (CERK) in the Golgi [3,69] (Figure 2). C1P may be converted back into ceramide by the action of one of LPPs or by C1P phosphatase (CPP) [3,69,70,71]. The irreversible reaction converting Cer to acylceramide (acylCer) is catalyzed by diacylglycerol acyltransferase-2 (DGAT2) [3] and is used to store Cer in lipid droplets [72] (Figure 2).

## 4. Overview of Major Functions of Bioactive Sphingolipids

Sphingolipids are constituents of cell membranes (about 50–60% of the membrane content) and important bioactive molecules acting as second messengers. They form lipid microdomains, important for cell signaling and participating in cell–cell interactions and absorption of extracellular lipids (e.g., fatty acids). SLs were shown to regulate calcium homeostasis, ROS formation, histone acetylation and activation of numerous transcription factors. Pathophysiological conditions result in alterations of the ratio between the structural and bioactive SL content, a phenomenon that has been shown to contribute to the regulation of the inflammatory response. Proinflammatory cytokines such as IL-1β and TNFα as well as several other stimuli (Fas ligand, phorbol esters, heat shock, oxidative stress and various chemotherapeutics) are well described disruptors of the SL homeostasis [3,54].

The best studied bioactive SLs are ceramide, sphingosine, S1P, C1P, as well as sulfatide. While ceramide and sphingosine are traditionally believed to exert proapoptotic signals, the action of S1P and C1P is largely context and tissue-dependent, with pro- and antiapoptotic activities described in various tissues. The intracellular concentrations of ceramides, sphingosine and S1P differ by an order of magnitude, with the lowest content of the least one. Therefore a small change in ceramide content might substantially impact S1P concentration. In the following sections the most important functions of major bioactive SLs in various cell types, which could be of relevance for beta-cell pathophysiology in T1DM, is briefly summarized. For a more detailed review on SLs in different tissues and disorders the reader is asked to refer to several excellent reviews addressing these topics [2,3,69].

### 4.1. Ceramide

Ceramide is implicated in a variety of cellular processes such as apoptosis, cell growth arrest, differentiation, cellular senescence, cell migration and adhesion [3,54,73]. Many proinflammatory mediators and oxidant agents have been shown to stimulate Cer generation, upon them T1DM-relevant cytokines (IL-1β, TNFα and IFNγ). The best described role of Cer is the regulation of apoptosis [3]. The main mechanism involved in Cer-induced apoptosis is the disruption of mitochondrial integrity and function [74,75]. The induction of mitochondrial apoptosis by ceramides was elegantly demonstrated by means of mitoCERT (CERT equipped with a MOM anchor), enabling Cer flow from ER to mitochondria. The translocation of ER ceramides to mitochondria resulted in the initiation of apoptosis [74]. Elevated levels of Cer were shown to control MOMP (mitochondrial outer membrane permeabilization) and thereby cytochrome c release, by fostering BAX/BAK oligomerization and formation of ceramide channels. Formation of Cer channels promotes the egress of proteins from the intermembrane space and ROS generation [74,75]. Cer contributes also to the suppression of mitochondrial electron transport chain and decreased ATP formation upon inflammation [76]. Recent studies identified the voltage-dependent anion channels VDAC1 and VDAC2 as mitochondrial ceramide binding proteins [77] and reported the expression of CerS within the mitochondrial compartment [77]. Moreover, the recent identification of a protein p17/PERMIT mediating ER-mitochondria tracking of newly translated CerS1 through MAMs to the mitochondrial outer membrane has been shown to be an event necessary for mitophagy induction [78]. CerS1/C18 ceramide generation was reported to foster LC3BII targeting of autophagolysosomes to mitochondria. Overexpression of CerS2, resulting in the formation of very long chain ceramides was also associated with mitophagy activation and mitochondrial dysfunction [78]. Furthermore, intramitochondrial accumulation of C16 generated by CerS6 was reported to induce mitochondrial fission and mitophagy (excellently reviewed by [73]). Additionally to these mitochondria-related effects, Cer was shown to regulate various signaling pathways (e.g., AKT/PKB or JNK) and to modulate different kinases (e.g., protein kinase C, PKC or protein phosphates PP1 and PP2A) [3]. Finally, elevated levels of Cer in the plasma membrane may increase membrane rigidity thereby stabilizing lipid rafts, which was shown to foster signal transduction [3,54].

### 4.2. Sphingosine

Similarly to Cer, sphingosine is thought to play an important role in cell cycle arrest and apoptosis [2,3]. Sphingosine was shown to inhibit the activity of PKC [2]. Moreover, sphingosine can bind to the anti-apoptotic protein 14-3-3 and inhibit its action by phosphorylation of the dimer interface [79]. Furthermore the ANP32a and ANP32b proteins have been shown to be targeted by sphingosine, and this is a mechanism by which sphingosine conveys its inhibitory effect on PP2A [80].

### 4.3. Sphingosine 1 Phosphate

Shingosine-1 phosphate is present in plasma (0.2–1 µM) and its concentration varies under different metabolic and pathological conditions. S1P is mostly bound to apolipoprotein M (ApoM, 50–70%) and albumin (ca. 30%), and recent reports point to an important role of HDL-bound S1P, especially in context of protection against cardiovascular disorders [81,82]. The main sources of blood S1P are erythrocytes and platelets [83]. The intracellular concentrations of S1P are much lower (nM, possibly due to a higher activity of enzymes involved in its turnover) and undergo severe changes under proinflammatory conditions. In most cell types S1P inhibits apoptosis, fosters cell proliferation and metabolism, and was shown to play a crucial role in immune cell trafficking and differentiation [1,2,84,85]. The deficiency of SPL promotes tumor growth and survival [86,87,88]. In neurons [89,90,91,92,93,94,95] and pancreatic beta-cells [21] intracellular S1P is involved in cytotoxic effects (see below). The effects of S1P are mediated by cell surface receptors and by activation of intracellular targets [1,2,3]. Five S1P receptors (S1PR1-S1PR5) were identified. They all belong to G protein-coupled receptors and are characterized by distinct mechanisms of action depending on the G subunit involved. Therefore the outcome of the receptor-dependent S1P action depends on the tissue-specific expression profile of S1PRs and its regulation upon various conditions [96]. Several proteins have been linked to cellular S1P export. Upon them the ABC transporter family and the Spns2 transporter are the best described [1,2,96]. Their cell type-specific expression determines S1P function in various tissues. The activation of S1PR2 was shown to stimulate cell-surface integrins and fibronectin matrix [96]. S1P has been shown to mediate the effect of cytokines on COX2 activation and PGE2 production [97]. Additionally to the classical receptors-dependent mediated S1P effects, the activation of intracellular target-dependent pathways participate in overall S1P action in cells. The zwitterionic head group of S1P, which is sufficiently water-soluble, enables S1P to flow between membranes and cellular organelles. Many molecules have been proposed as intracellular targets of S1P, among them the CIAP2 (cellular inhibitor of apoptosis 2), CerS2 and TRAF2 (TNF receptor associated factor 2) proteins [1,2,3,98]. Of note, the direct involvement of S1P in the regulation of TRAF2 activation was questioned by Etemadi and coworkers [99]. Moreover the SK2-mediated formation of S1P in the nucleus has been associated with changes in the epigenetic signature of cells due to the ability of S1P to directly bind and inhibit the histone deacetylases HDAC1 and HDAC2 [100,101]. Finally, many studies reported calcium as a possible second messenger in the intracellular action of S1P [21,89,93].

### 4.4. Ceramide-1 Phosphate

The function of ceramide 1-phosphate, similarly to S1P, is largely tissue and cell-context specific, with anti- and proinflammatory properties [70,102]. The enzymatic activity of CERK was shown to be upregulated by IL-1β, macrophage colony stimulating factors (M-CSF), calcium ionophore A23187, tyrosine-kinase pathway and agonists of nuclear peroxisome proliferator activated receptors (PPARs) [69]. C1P is believed to have strong mitogenic and antiapoptotic activities [69].

### 4.5. Sulfatide

Sulfatide (3-O-sulfogalactosylceramide) is a glycoSL that is synthesized from Cer by two transferases (ceramide galactosyltransferase and cerebroside sulfotransferase). Sulfatide is a multifunctional bioactive SL, which was demonstrated to play an important role in the nervous system, pancreas, immune system, as well as bacterial and virus infections (for more details please refer to [103]. Sulfatide is present predominantly in the nervous system and pancreatic islets [104]. The enzyme arylsulfatase A (ASA) specifically degrades it [103], though an ASA-independent degradation of sulfatide was also described. ASA activity requires the help of saposin B, which liberates sulfatide from membranes and makes it accessible for ASA [103]. Sulfatide is localized mainly in the Golgi apparatus, cellular membrane, and lysosomes; in pancreatic beta-cells it was found in insulin granules [103]. Moreover, sulfatide can activate binding of laminin to integrins and was described as a major L-selectin ligand, playing an essential role in monocyte infiltration in some organs [103].

## 5. Effects of Proinflammatory Cytokines on the Sphingolipid Pathway Enzymes in Pancreatic Beta-Cells

Most data available regarding the sphingolipid pathway in pancreatic beta-cells has been gained from murine and rodent models (RINm5F, INSE cells, MIN6 cell lines and mouse models). The extensive qRT-PCR analysis revealed the presence of all enzymes involved in the SL pathway in rodent beta-cells [21]. Pancreatic beta-cells are characterized by an imbalance of the enzymatic capacity for S1P formation (SK1 and SK2) and degradation (SPL) [21], which seems to play a particularly important role for cytokine toxicity (see Section 7).

Although a growing number of studies describing changes in the SL content upon T1DM development in humans are available, the SL rheostat of human beta-cells has not yet been characterized. Moreover detailed data about the expression pattern of the SL pathway enzymes in beta-cells in animal models of T1DM and in the human pancreas from T1DM individuals is still missing.

### 5.1. De Novo Synthesis

The enzymes catalyzing the first steps of the SL biosynthesis are particularly sensitive to cytokine action in rat insulin-secreting INS1E cells [21]. The subunits of SPT are affected differentially, namely the long chain 1 and long chain 2 subunits are strongly increased in response to proinflammatory cytokines (IL-1β, TNFα and IFNγ), while no changes in the expression level of the short chain subunit were detected [21]. Cytokines significantly stimulate the expression of DES in rat INS1E cells [21]. Interestingly, data from human islet transcriptome revealed that ORMDL3 is expressed in human islets and undergoes upregulation upon 24 h-exposure to a mixture of IL-1β and IFNγ [32]. Whether this expression can be accounted to beta-cells, what the contribution of TNFα on the expression of ORMDL3 is and if beta-cells express also other isoforms of ORMDL proteins requires further investigations. The ORMDL3 gene polymorphism was described in the recent studies involving T1DM individuals [14,105].

Recently a new mechanism regulating the expression of SPT has been described [106]. Using genome-wide CRISPR/Cas9 screening it was demonstrated that AHR (aryl hydrocarbon receptor) binds and activates the gene promoter of SPT [106]. Moreover, tissues from AHR KO mice were characterized by reduced expression of several other key genes in the SL biosynthetic pathway and decreased the SL content [106]. Interestingly, AHR is a transcription factor that has been recently shown to link the diet and gut microbiome alterations with islet autoimmunity [107], yielding the possibility that the observed activation of AHR in islets during T1DM development could stimulate the SPT expression and increase de novo SL biosynthesis in beta-cells. Whether this is indeed the case still needs to be experimentally proven.

### 5.2. Ceramide Metabolism Regulation

The best studied part of the SL biosynthesis pathway in beta-cells is the formation of Cer. IL-1β was the first cytokine shown to activate the sphingomyelin/ceramide pathway in rat insulin-producing RINm5F cells [108]. The studies revealed that a short exposure to IL-1β (2–5 min) could induce Cer and diacylglycerol (DAG) generation with a parallel rapid decrease of sphingomyelin content, indicating the activation of sphingomyelinase [108]. Another report evaluated the sphingomyelin hydrolysis in response to IL-1β exposure in rat and mouse islets as well as in RINm5F cells and failed to detect a significant effect of IL-1β within the time-frame of the experiment [109]. The elevation of Cer content in beta-cells in response to TNFα was demonstrated in mouse insulin-secreting MIN6 cells and it was suggested to be involved in TNFα-mediated apoptosis [110]. These observations were not confirmed in another insulin-secreting mouse cell line, the β-TC3 cells, in which cytokines (IL-1β, TNFα and IFNγ) failed to stimulate Cer formation [111]. The sphingomyelin content was around 50% decreased in β-TC3 cell line, similarly to RINm5F cells, however the authors showed that the kinetics of sphingomyelin hydrolysis within 4 h after cytokine addition did not differ between control and cytokine-treated cells [111]. The reasons for the contradictory findings could be related to methodological limitations of these early studies, time-specific effects of cytokine action and/or caused by a quick turnover of SLs in beta-cells.

More recent studies, involving modern mass spectrometry technology, confirmed an induction of neutral SMase and a parallel activation of iPLA2β leading to Cer accumulation in rat insulin-secreting INS1 cells exposed to proinflammatory cytokines (IL-1β + IFNγ) [112]. Our studies in INS1E cells revealed that also the mRNA expression of acid SMase is upregulated in response to a mixture of cytokines (IL-1β, TNFα and IFNγ) [21]. Beta-cell specific overexpression of iPLA2β in a mouse model (RIP-iPLA2β-Tg mice) results in upregulation of nSMase mRNA and protein expression, followed by decreased sphingomyelins with a parallel increase of Cer content [113]. This was accompanied by ER stress, mitochondrial damage and caspase-3 activation [112,113].

Ceramide content can also be upregulated by stimulation of Cer production by CerS. We showed that in beta-cells proinflammatory cytokines upregulate the mRNA expression of various types of CerS [21], strongly supporting a notion that under T1DM conditions an intra-beta-cell ceramide formation might be indeed induced. The most prevalent CerS isoforms in rodent beta-cells are CerS2 and CerS5/6, followed by CerS1 ([21], and Gurgul-Convey, unpublished). These isoforms are characterized by distinct subcellular localization (ER vs. mitochondria), as well as differences in the length of generated ceramides. Owning the role of ER stress and mitochondrial damage in cytokine toxicity to beta-cells, the cytokine-mediated induction of CerS expression may significantly contribute to toxic effects of cytokines to beta-cells. An increased Cer generation may particularly promote cytokine-induced mitochondria damage in beta-cells. The recent findings from the Brüning group strongly indicate such a scenario [114]. Using the CerS6 deficient mouse model the authors demonstrated that CerS6-derived C16 Cer, in contrast to CerS5-derived Cer, could promote mitochondrial fission and insulin resistance in obesity [114]. Using the CerS6 KO model exposed to STZ-mediated islet autoimmunity could enable in the future to assess the importance of mitochondrial Cer formation in the development of T1DM.

The involvement of mitochondrial ceramide in cytokine toxicity is indirectly suggested also by our observations in SPL-overexpressing INS1E cells [21]. Recently, we showed that the overexpression of SPL in beta-cells protects from cytokine toxicity by prevention of the ER-calcium leak, decreased mitochondrial calcium levels and promoting the expression of mitochondrial chaperones [21]. SPL deficiency was associated with increased Cer levels in other cell types [115]. Though the effects of SPL overexpression on Cer content in beta-cells still need to be analyzed, it seems plausible that the observed beta-cell protective effects of SPL overexpression may be at least partially related to decreased Cer levels, particularly in the mitochondrial compartment.

Another interesting aspect of mitochondrial Cer formation in the context of beta-cell fate in T1DM could involve sirtuins. Sirtuins are NAD+-dependent histone/protein deacetylases consisting of several subtypes that are present in various subcellular compartments and are involved in the regulation of oxidative stress through direct deacetylation of transcription factors controlling antioxidant genes [116]. Additionally, sirtuins are believed to be important regulators of glucose homeostasis, insulin secretion and mitochondrial biogenesis. Interestingly, the mitochondrial SIRT3 has been recently shown to regulate Cer generation in the brain in response to ischemia/reperfusion [76]. So far the role of altered expression of sirtuins, on mitochondrial formation of Cer in beta-cells has not been investigated.

Finally, cytokines were reported to induce the activity of aCD in INS1 cells and primary rat islets [117]. The pharmacological inhibition of aCD by NOE resulted in enhanced cytokine toxicity in INS1 cells [118]. In contrast to neutral CD [118], no changes on the mRNA and protein levels of aCD in INS1 cells and rat islets treated with cytokines were detected for up to 4 h [117]. We extended this study to a more mature beta-cell line, namely INS1E, and analyzed the mRNA expression pattern of acid and neutral CDs under longer time periods [21]. Our measurements revealed that a mixture of three major proinflammatory cytokines induce the upregulation of both nCD and aCD in INS1Ecells [21].

### 5.3. S1P Metabolism, Receptors and Transporters

We observed similar effects of high concentrations of IL-1β (as prevails in the first stage of islet autoimmunity) and of a mixture of the three major diabetogenic cytokines IL-1β, TNFα and IFNγ (as it occurs in the advance stage of insulitis) on the mRNA expression level of enzymes involved in S1P metabolism, indicating a crucial role of IL-1β [21]. This was in contrast to distinct effects of the acute (6 h) versus prolonged (24 h) cytokine exposure on the gene expression of enzymes involved in the SL metabolism, S1P receptors and S1P transporters [21].

Early reports identified four types of S1P receptors (S1P1, S1P2, S1P3 and S1P4) in mouse and rat islets, and in INS1 cells [119]. Recently we demonstrated the mRNA expression of S1PR2, 3 and 5, of which S1PR3 was the predominant subtype, in INS1E beta-cells [21]. S1PR2 and S1PR3 are coupled predominantly to Gq and activate phospholipase C (PLC) to induce Ca^2+^ mobilization through the production of inositol 1,4,5-trisphosphate [120,121], and induce activation of MAPK kinases [122]. S1PR5 was shown to interact with Gα subunits [123], which inhibit PLC activity. Beta-cells are equipped with various S1P transporters [21]. The Abca1 transporter is expressed at the highest level, followed by Abcc1 and a nearly 100-fold lower expression of Spns2 [21]. Acute exposure to proinflammatory cytokines results in a strong downregulation of S1PR3 (70% decrease), partially compensated by a mild upregulation of S1PR2. S1PR5 remains unchanged under such conditions [21]. Upon longer incubation with cytokines, the expression of all S1P receptors undergoes upregulation [21]. Similar observations were made in the case of S1P transporter mRNA expression [21]. The impact of these alterations of S1P receptor and transporter system in beta-cells under acute and chronic cytokine exposure on beta-cell fate has not yet been investigated, but it could provide interesting insights into the role of S1P in beta-cell vulnerability to cytokines.

Proinflammatory cytokines enhance the activity of SK in rat beta-cells [124,125]. Our gene expression data suggest that SK2 is the main isoform expressed in insulin-secreting cells and proinflammatory cytokines increase its expression [21]. Cytokines downregulate the expression of SPL in INS1E cells and rat islets, while they enhance the expression of SPP2 [21]. These observations suggest an increased rate of sphingosine generation in beta-cells upon cytokine exposure. This could foster increased Cer formation and/or accumulation in mitochondria or elevate the generation rate of S1P in mitochondria, nucleus und other specific locations. Since SPP has not yet been shown to localize in mitochondria and nucleus, the cytokine-induced S1P formation in these two subcellular compartments is likely unlimited in contrast to other compartments with a parallel cytokine-mediated overexpression of SPP. Thus cytokine action could affect subcellular SL gradients in beta-cells with high local Cer, sphingosine and/or S1P concentrations upon cytokine exposure.

Finally, the cytokine-mediated downregulation of SPL, which we observed in INS1E cells, does not coincidence with increased S1P concentration (Gurgul-Convey, unpublished). This observation further suggests a shift of the SL pathway to sphingosine/Cer formation in beta-cells exposed to cytokines. Such a phenomenon was observed in the Charcot–Marie–Tooth phenotype in humans that is characterized by SPL deficiency and was shown to be associated with elevated Cer levels [126,127].

How these changes on the expression level of enzymes of the SL pathway translate to the sphingolipid composition of beta-cells exposed to cytokines should be further investigated, since rearrangements of the beta-cell SL profile may have potentially remarkable consequences on the susceptibility of beta-cells towards proinflammatory cytokines. This could include alterations of the expression pattern of HLA Class I and II, acceleration of cytokine signaling or disturbed cell–cell interactions. Additionally an interesting question arises whether intracellular S1P may participate in the epigenetic regulation of genes relevant for beta-cell vulnerability to the autoimmune reaction in T1DM. Further studies are needed to characterize the changes of the SL enzymatic machinery in human beta-cells before and after T1DM onset.

## 6. Effects of Bioactive Sphingolipids on the Beta-Cell Function in T1DM

Multiple studies have demonstrated that various SLs may regulate the beta-cell secretory capacity [4,8,9,128,129,130]. These effects are conveyed by the activation of cell surface receptors, a regulation of ion channels or intersection with insulin production and folding. The abundance of sphingomyelin patches on beta-cell surface was additionally reported to modulate the insulin secretory capacity [131].

The inhibitory effects of Cer and its analogues on insulin production and secretion are well described [111,130,132,133]. So far no data is available on the effects of intracellular Cer production topology on GSIS disturbances in cytokine-treated beta-cells. In contrast to Cer, extracellular S1P is a potent stimulator of insulin secretion [21,129]. Upon the activation of its receptors, S1P potentiates GSIS, most likely by induction of cAMP generation [21,125]. Deletion of SK1 in INS1E cells results in defective insulin gene expression, lower insulin content and GSIS [134]. SK2 KO in MIN6 cells leads to higher GSIS, also in the presence of low glucose [129]. Our studies showed that depletion of intracellular S1P content by overexpression of SPL does not affect GSIS in the absence of proinflammatory cytokines [21]. SPL overexpression was however capable to partially protect against proinflammatory cytokine-mediated GSIS inhibition [21]. While the exact mechanism underlying this protective effect of SPL overexpression needs further investigation, we observed an increased protein expression of mitochondrial chaperones, which play an important role in ATP synthesis (mimitin and prohibitin 2) [21]. Earlier studies revealed that these mitochondrial proteins are essential for a proper GSIS [42,135]. siRNA-mediated inhibition of Phb2 in SPL-overexpressing beta-cells resulted in a partial loss of SPL-mediated protection [21]. Additionally, we observed an increased expression of BIP and Sec61a in ER of SPL-overexpressing INS1E cells that correlated with improved calcium homeostasis [21]. BIP and Sec61a cooperate to prevent a calcium leak from ER [136]. Therefore their enhanced expression in SPL-overexpressing INS1E cells could prevent cytokine-mediated disruption of calcium homeostasis. Moreover, improved BIP expression in SPL-overexpressing INS1E cells may help to secure unbiased insulin folding in cytokine-treated cells.

In the first hours of IL-1β action, which is not associated with cytotoxicity, a temporary potentiation of GSIS is observed [137]. Could cytokine-induced S1P generation be used by beta-cells for promoting this effect? In contrast, the acute phase of cytokine toxicity is accompanied by dysregulation of GSIS [21,51,52,53]. In this acute phase of cytokine toxicity the expression of SK2, S1P transporters and receptors expression is upregulated while SPL expression is downregulated. This is likely to result in decreased intracellular levels of S1P, due to a parallel high expression of SPP and CerS [21]. Since SK2 is not expressed on the plasma membrane, the SK2-derived S1P is not expected to be efficiently transported inside-out and activate its receptors on the beta-cell surface. Therefore it seems that the increased expression of S1P transport and the signaling system in beta-cells could serve as an adaptation strategy to make up for cytokine-mediated inhibition of GSIS by S1P-mediated cAMP generation and its potentiating effect on GSIS. Further studies are needed to describe the effects of cytokines on the SL content upon an acute phase of cytokine toxicity and the chronic incubation. The results may help to understand the natural history of disturbances of beta-cell function in early and advance stages of cytokine toxicity and could enable designing preventive strategies.

Another twist to SL effects on beta-cell function was added by demonstration that synthaxin 4 (Stx4) is required for aSMase activity [138]. Stx4 is a plasma membrane–localized exocytosis protein that is crucial for GSIS [139]. Interestingly, Stx4 is a T1DM candidate protein [140]. Could therefore Stx-4-mediated changes in the beta-cell sphingomyelin content be involved in beta-cell dysfunction upon cytokine exposure?

Probably the best studied complex glycoSL in context of insulin production and secretion is sulfatide [103]. Interestingly, in T1DM patients the content of sulfatide was recently shown to be significantly reduced [14]. In pancreatic beta-cells sulfatide is present at the surface membrane of and in secretory granules [141], with a predominantly expressed C16:0 isoform [14]. Sulfatide is believed to promote proinsulin folding and to serve as a molecular chaperone for insulin [142]. The content of sulfatide in insulin granules decreases with rising metabolic activity of beta-cells [14]. Sulfatide is secreted together with insulin, facilitates rapid insulin monomerization and is crucial for insulin crystal preservation [142]. Moreover, sulfatide is required for normal insulin secretion through the activation of ATP-sensitive potassium ion channels and stimulation of calcium ion-dependent exocytosis [143]. In vivo administration of Zucker rats with C16:0 sulfatide resulted in significantly elevated GSIS without effects on glucose tolerance [144].

These results suggest that the place of origin and the type of SLs might be crucial for the final outcome of particular SLs on the beta-cell function. Further studies are needed to characterize which types of SLs are generated upon exposure to diabetogenic cytokines and how they influence insulin biosynthesis and beta-cell secretory capacity under T1DM conditions. The use of islets isolated from SK1, SK2 and SPL KO mouse models and exposed to cytokines will be useful in this context.

## 7. Effects of Bioactive Sphingolipids on Beta-Cell Fate in T1DM

Accumulative data indicates that SLs may be crucially involved in beta-cell death during T1DM development. Though the exact underpinning mechanisms remain unclear, evidence indicates that two elements may be of particular importance, namely the changes of the SL pattern of beta-cells and alterations of SL profiles in islet surroundings.

Toxic effects of Cer and sphingosine on beta-cells are well documented [4,5,110,111,118,130,133,145]. As discussed in the Section 5, proinflammatory cytokines were found to induce Cer formation, which was associated with apoptosis. Interestingly, overexpression of Stx4, which was shown to stimulate aSMase activity, reduces the chemokine ligand gene expression in beta-cells and protects beta-cells against cytokine toxicity [139]. Stx4 is a T1DM candidate gene [140]. Therefore a question arises whether the Stx-4-mediated changes in the beta-cell SL rheostat could be a link to islet autoimmunity by affecting cell membrane composition and thereby reducing the chemokine ligand presence on the beta-cell surface.

The role of S1P in cytokine toxicity to beta-cells has been extensively studied in the last decade. First it was demonstrated that extracellularly added S1P protects insulin-secreting INS1 cells and rat islets against cytokine toxicity [124,125]. The cytokine-mediated TUNEL staining, cytochrome c release and caspase-3 activation were reduced after treatment with nM concentrations of S1P. The S1P receptor antagonist BML-241 blocked this protective effect. Beneficial effects of S1P against cytokine toxicity were not associated with decreased cytokine-mediated iNOS expression or NO generation. This observation is particularly interesting in the context of human beta-cells, in which cytokines fail to induce the iNOS pathway and are nevertheless toxic [42,146]. How would extracellular S1P protect the beta-cell against cytokine toxicity? Laychock and colleagues showed that exposure of beta-cells to S1P leads to the stimulation of PLC activity, indicating the activation of Gq subunit of S1PRs in beta-cells [125]. Our own studies revealed that exposure of INS1E cells to S1P results in a rise of cAMP generation [21], extending the earlier observations that S1PR2 activation induces cAMP generation in other cell types [147,148,149,150]. Since cAMP conveys its cytoprotective effects in beta-cells via multiple mechanisms, including PKA activation and regulation of calcium homeostasis [40,151], it would be worth evaluating these pathways in detail by means of specific inhibitors and/or genetic modifications of S1P metabolism.

Could the addition of S1P to the culture medium for islets isolated for transplantation be implemented as a preservation method? Against this procedure points the fact that extracellular S1P was reported to increase insulin secretion, also in the absence of hyperglycemia [21]. Such a scenario could lead to depletion of beta-cell insulin capacity and lead to metabolic stress of isolated islets, making islets bathed in a S1P-containing medium less useful for transplantation. This is in contrast to another bioactive lipid compound, namely prostacyclin and its analogues, which also stimulate cAMP generation in beta-cells, however do not potentiate insulin secretion in the presence of low-glucose culture medium [151]. Furthermore, S1P has been shown to be implicated in islet allograft survival [152,153]. Fingolimod, a S1PR modulator, was demonstrated to enable long-term survival of islet allografts due to its effects on immune cell trafficking [152,153].

Interestingly, the action of intracellularly generated S1P in cytokine-treated beta-cells seems to be opposite to that of extracellular S1P [21]. Our data strongly indicates that intracellularly generated S1P participates in acute cytokine toxicity to beta-cells [21]. We observed an intermediate mRNA expression level of SPL in rodent beta-cells and islets as compared to other tissues [21]. This was downregulated in response to cytokines [21]. Overexpression of SPL protected insulin-secreting cells against cytokine-induced apoptosis [21]. SPL overexpression was accompanied by maintenance of calcium homeostasis, which is strongly impaired by the action of proinflammatory cytokines in beta-cells [21]. Additionally, SPL-overexpressing INS1E cells were protected against cytokine-mediated ER stress, as evident by a significant inhibition of CHOP expression after incubation with cytokines. Moreover, SPL overexpression reduced cytokine-mediated inhibition of cell proliferation and ATP content [21]. These protective effects were independent from the NFκB-iNOS pathway [21]. Furthermore, SPL overexpression provided protection against cytokine toxicity though it failed to downregulate cytokine-induced ROS generation. As mentioned above, we detected a higher expression of various ER and mitochondrial chaperones in SPL overexpressing INS1E cells, indicating that changes in intracellular S1P concentrations may indeed epigenetically regulate gene expression in beta-cells, like in other cell types [100,101]. Importantly, siRNA-mediated suppression of SPL expression resulted in opposite effects to those observed in SPL overexpressing INS1E cells [21]. Interestingly, though SPL overexpression has been reported to be implicated in toxic effects of hexadecenal accumulation in various cell types [62,63], in beta-cells SPL overexpression provided protective effects. The possible explanation to this phenomenon could be that beta-cells are rich in the enzyme responsible for hexadecenal detoxification, namely ALDH3A2 [21], enabling prevention of hexadecenal accumulation and toxic effects of SPL-overexpression in beta-cells. It will be important to evaluate the expression and activity of SPL in beta-cells chronically exposed to cytokines, and to investigate the effects of double-transfection approaches including SK1/SK2 and SPL to determine the role of intracellular S1P in more detail.

The involvement of intracellularly generated S1P in cytotoxic effects of cytokines in beta-cells seems to be very similar to the role of intracellular S1P in neurons as described in elegant studies by the Van Echten-Deckert group [89,90,91,92,93,94,95]. Interestingly, pancreatic beta-cells and neurons share multiple common features, though derived from distinct germ layers [154]. It is speculated that the endocrine and nervous systems developed from a common evolutionary ancestor [155,156]. Moreover, *Drosophila*-insulin-like peptides (Dilps), which are synthesized by neurons in flies, were shown to regulate energy metabolism similarly to mammalian insulin [157]. The way beta-cells biosynthesize and store insulin, and answer to external stimuli by insulin secretion mimics very closely the way neurons store and release neurotransmitters [154,156]. Many studies showed that beta-cells and neurons are characterized by similar gene expression patterns and spliceosome activity [158] (for more information please refer to [154]). It is therefore not surprising that beta-cells and neurons may also share similar sensitivity to intracellular S1P.

The mRNA expression of both SPP1 and SPP2 was found to be induced in beta-cells under acute exposure to cytokines [21]; what the impact of a chronic exposure to cytokines on SPP expression is or whether the expression/activation of other phosphatases would be of importance for beta-cell fate should be addressed in the future. Though exogenous Cer was shown to disrupt mitochondrial function [145] and induction of SMase was shown to be accompanied by increased Cer content, mitochondria damage and apoptosis [112], there is no direct evidence linking Cer accumulation in mitochondria to cytokine-mediated beta-cell apoptosis. Finally, several SL-enzyme knockout mouse models have been successfully characterized in context of T2DM [159,160,161] and many of them show a phenotype that could be interesting for T1DM research. For example mice lacking SPP2 display defective beta-cell proliferation, reduced islet mass and ER stress activation in beta-cells [159]. Exposure of such animal models to STZ, generation of beta-cell specific SPL knockout and knockin mouse models or development of SL-enzymes knockouts in Non Obese Diabetic (NOD) mice, will advance our understanding of T1DM development mechanisms.

Another interesting aspect of cytokine action on the SL pathway is the secretion of nCD via exosomes. Interestingly the low nontoxic cytokine concentrations [38,162] were shown to stimulate nCD, in a similar manner to high concentrations [163]. The lack of toxic effects of low-dose cytokine exposure correlated with a release of neutral ceramidase via exosomes [163] and the presence of nCD containing exosomes prevented apoptosis in INS1 cells incubated with high concentrations of cytokines [163]. The generation and secretion of S1P via nCD-rich exosomes was responsible for the activation of S1PR2 and the observed antiapoptotic effect. This study indicates that beta-cells may activate protective mechanisms at the beginning of the inflammatory response within islets, to prevent further damage caused by high concentrations of cytokines. Once this axis fails, the beta-cell apoptosis and destruction are accelerated.

Overall, this data indicates that proinflammatory cytokines may impact SL generation differentially upon acute and chronic exposure that could have potentially pronounced consequences for beta-cell viability and vulnerability to autoimmune insult. Future measurements of SL species and their distribution in cytokine-treated beta-cells should help to understand how these observed effects influence beta-cell vulnerability to cytokines.

## 8. Sphingolipids in Animal Models of Autoimmune Diabetes and Human T1DM

In this chapter the SL changes occurring in animal models of autoimmune diabetes and in human T1DM and their role for islet autoimmunity and for beta-cell survival will be addressed. As discussed above proinflammatory cytokines that are secreted by activated immune cells infiltrating islets during T1DM development were shown to affect the SL metabolic pathway of beta-cells. Changes in the SL content were associated with beta-cell dysfunction and death. Recent studies demonstrated an altered SL profiles in the blood of T1DM patients [14,15,16,18,130,164]. What are the consequences of these altered SL serum profiles on pancreatic beta-cell function and fate during T1DM development? Is the SL concentration around the islets altered? Is the SL composition of beta-cell distinct in T1DM individuals comparing with nondiabetic, healthy subjects?

### 8.1. Animal Models

While these questions remain at present open, the studies with the S1P receptor modulator Fingolimod/Gilenya (FTY720) may shed some light on this topic. Fingolimod was approved for the treatment of multiple sclerosis in over 40 countries [165]. Its effects were analyzed in multiple animal models of autoimmune diabetes, such as the NOD mouse, STZ-induced autoimmune diabetes in mice and in the rat model of human T1DM, the LEW.1AR1-iddm (IDDM) rat [166,167,168]. In all these animal models fingolimod treatment was reported to improve glycemia and prevent infiltration of islets. Long-term treatment with FTY720 proved to prevent the diabetes onset in IDDM rats and in NOD mice by reducing immune cell infiltration and cytokine-mediated beta-cell destruction [152,153,166,167,168].

The IDDM (LEW.1AR1-iddm) rat develops spontaneously autoimmune diabetes [169]. Pancreatic islets from fingolimod-treated IDDM rats are characterized by a well preserved architecture and dense insulin immunostaining [167]. This goes along with prevention of T-cell islet infiltration and reduced expression of proinflammatory cytokines in immune cells [167]. The remaining weak macrophage infiltration observed in the minority of islets in fingolimod-treated IDDM rats correlates with the increased beta-cell expression of MCP1, a well-known attractant for macrophages [167]. Interestingly, the infiltrating macrophages in fingolimod-treated IDDM rats do not express and release IL-1β and TNFα even after a prolonged time post fingolimod treatment, explaining the lack of beta-cell demise in these islets. The study by Jörns and colleagues was the first to show that fingolimod treatment may result in decreased expression of proinflammatory cytokines. Why did macrophages remain proinflammatory cytokine negative in response to the fingolimod treatment? Jörns and colleagues observed that the treatment with fingolimod leads to increased production of anti-inflammatory cytokines IL-4 and IL-10 [167], which leads to the activation of an anti-inflammatory M2 phenotype [170,171]. IL-4 was also shown to protect beta-cells against cytokine toxicity [38,172,173,174]. Thus the phenomenon of fingolimod-mediated macrophage phenotype rearrangements opens a new intriguing research area in the S1P biology.

Further insights into the role of SLs in T1DM development were gained in a well-established mouse model of autoimmune diabetes, the NOD mouse. The NOD mouse is characterized by a genetic susceptibility to autoimmune diabetes and spontaneously develops autoimmune reaction against pancreatic beta-cells. Environmental factors have also been shown to affect the diabetes incidence in this mouse model [175]. A continuous supplementation of L-serine, a precursor of the SL biosynthesis, was shown to reduce diabetes incidence and insulitis score in female NOD mice [176]. The authors observed significant changes in serum SLs, failed however to detect any significant effects on the pancreatic SL content. Such effects of L-serine should not, however, be excluded since the authors did not perform experiments in purified beta-cells. Administration of fingolimod prolonged the survival rate of islet allografts in diabetic mice [152,153]. Another study showed that the serum phospholipid and triglyceride composition might be associated with progression to T1DM both in NOD mice and humans [164]. The authors found that young female NOD mice who later progress to autoimmune diabetes exhibited the same lipidomic pattern as prediabetic children [164], confirming that the NOD mouse is a valuable model to study the role of SLs in T1DM development. Moreover, NOD thymocytes were found to be characterized by a lower level of S1PR1 and a decreased SPL mRNA and protein expression comparing with healthy mice [177]. These changes were suggested to participate in the T-cell migratory abnormalities observed in NOD mice during diabetes development [177]. S1P was shown to reduce CD4+ T-cell activation in NOD mice and to prevent vascular complications [178].

Administration of glycoSLs has been widely used to assess their effects on islet autoimmunity in NOD mice. For instance, ganglioside GM1 decreased the rate of islet infiltration, attenuated production of proinflammatory cytokines (IL-1β, TNFα and IFNγ) and increased the level of NGF in islets [177]. The protective effects of sulfatide against autoimmunity have been recognized already many years ago when Buschard and colleagues demonstrated that the treatment with sulfatide or its precursor GalCer prevents diabetes in NOD mice [179]. Sulfatide was reported to increase the population of CD3+CD25+ regulatory T-cells, while decreasing production of proinflammatory cytokines (for details refer to the excellent review [104]). These protective effects of GalCer and sulfatide against autoimmune reaction during T1DM development rely on their effects on NKT) cells. In T1DM individuals NKT cells are less frequent and display deficient IL-4 responses [180]. Similar observations were made in NOD mice [181]. Interestingly, α-GalCer was shown to activate NKT cells and prevent the onset and recurrence of T1DM in NOD mice [181]. Moreover, a sphingosine truncated derivative of α-GalCer, OCH, was reported to prevent insulitis and diabetes development in NOD mice more efficiently than its precursor, probably by enhancing the activity of NKT cells to produce IL-10 [182]. Rhost and coworkers observed that a fraction of NOD mice develop autoantibody reactivity to sulfatide, though they failed to demonstrate that sulfatide treatment reduces the diabetes incidence under the treatment scheme they undertook [183]. Using fenofibrate, which activates the sulfatide biosynthesis, Holm and colleagues were able to completely prevent diabetes development in NOD mice [14]. In the follow-up study they demonstrated that fenofibrate selectively elevates the pancreatic content of very-long-chain SLs in NOD mice and reduces the incidence of diabetes by around 50% [184]. Moreover they showed that fenofibrate treatment leads to remodeling of pancreatic lipidome with increased amount of lysoglycerophospholipids [184]. NOD mice treated with fenofibrate were characterized by more stable blood glucose and improved glucose tolerance [184]. Sulfatide was additionally shown to inhibit insulitis and to prevent diabetes in NOD mice by blockage of L-selectin [185]. Furthermore, the C16:0 isoform of sulfatide was reported to downregulate the production of proinflammatory cytokines [186]. In vitro experiments revealed that sulfatide has also the ability to reduce caspase-3/7-dependent apoptosis caused by exposure of insulin-secreting cells to IL-1β, IFNγ and TNFα [187].

Finally, in two additional animal models of autoimmune diabetes (STZ-induced diabetic rats and Akita diabetic mice) the serum concentration of S1P was shown to be significantly elevated in comparison to control animals [17], raising a question whether following the S1P content in blood could serve as a biomarker to track the disease progress.

### 8.2. Human T1DM

The incidence of T1DM is rising in the last decades in western countries; this suggests an important role of environmental factors in the pathogenesis of this disease. Accumulating evidence points to significant changes in serum metabolome proceeding T1DM development [14,15,18,130,164]. An increased risk of T1DM was described in response to prenatal exposure to perfluoroalkyl substances that modulate neonatal serum phospholipids [188]. Several phospholipids, particularly sphingomyelins and specific phosphocholines, which were shown to be significantly lower in the serum of children who later progress to T1DM [18] were downregulated by perfluoroalkyl substances [188]. Holm and colleagues identified polymorphisms in eight genes encoding proteins involved in the SL metabolism that contribute to the genetic predisposition to T1DM [14]. Single-nucleotide polymorphisms (SNPs) in the chromosome 17q12-q21 region with the gene coding for ORMDL3 were linked with T1DM [14,105]. Importantly, the level of these polymorphisms correlated with the degree of islet autoimmunity in patients with recent onset T1DM [14].

Interestingly alternations of S1P levels have been shown to control IL-17 production in human T-cells [189]. IL-17 arises as a crucial player in the autoimmunity development in T1DM [190,191]. Therefore a control of S1P levels and of the activation of S1PRs might represent an interesting intervention option that should be tested in the future.

With the respect to S1P action, it is important to mention that the cardioprotective effects of HDL have been shown to depend on its chaperoning function for S1P [81,82]. Dyslipidemia is typical in patients with long-term T1DM, and associates with an increased risk of cardiovascular events. How sphingolipids, or particularly S1P, contribute to the progression of diabetic complications in T1DM-patients requires further investigations.

Furthermore, an altered SL content in immune cells, peripheral blood mononuclear cells (PBMC) as well as in serum were reported in multiple studies with T1DM individuals [14,15,18,130,164]. The changes in the sphingomyelin content were defined as a new hallmark of progression to T1DM. Moreover sulfatide levels in newly onset of T1DM patients were found significantly lower than in healthy children [14]. Studies performed in the newborn infants, who later in life progress to T1DM, indicate that their lipidomic profiles are distinct from those of healthy infants [19,20]. The recently published clinical case report study by the Buschard group demonstrated that the treatment with fenofibrate (160 mg daily) initiated seven days after T1DM diagnosis resulted in a fast decline of insulin dose and long-term insulin-independency [192].

The ceramide pathways were found to be specifically associated with T1DM progression [18]. In T1DM progressors a lower content of L-serine was described, in line with the protective effects of L-serine administration against islet autoimmunity observed in the NOD mouse [176]. This was in contrast to glycoSLs such as GlcCer, LacCer and GalCer, which were significantly upregulated in T1DM patients. The authors concluded that these changes in the SL blood cell content could contribute to immune dysfunction in children, who later progress to T1DM [18].

Finally, an intriguing observation about a protective role of C1P on insulin signaling in diabetic kidney [193] might also apply to the phenomenon of insulin resistance in T1DM [194]. SMPDL3b (sphingomyelin phosphodiesterase acid-like 3b) is a lipid raft enzyme that regulates plasma membrane fluidity. Its enhanced expression was observed in diabetic kidney disease and was shown to affect the production of SLs resulting in decreased C1P content [193]. The SMPDL3b overexpression impaired insulin signaling by interfering with insulin receptor isoforms binding to caveolin-1 in the plasma membrane, which was rescued by supplementation with exogenous C1P [193]. Moreover S1P was shown to counteract insulin signaling in beta-cells through the activation of S1PR2 [128]. These studies are raising the question whether a lipid-based therapy might be an option for treatment of severe T1DM complications. Further studies involving human beta-cells, genetically modified beta-cell lines, and various T1DM animal models are needed to assess the role of C1P in T1DM development and beta-cell fate.

## 9. Sphingolipids as Autoantigens and Biomarkers in T1DM

The progression to T1DM is monitored mainly by evaluation of serological biomarkers (autoantibodies). The positivity of autoantibodies against beta-cells, the age of seroconversion and the positivity for multiple autoantibodies are predictors and major risk factors for the development of T1DM [195,196,197]. The primary islet autoantibodies are autoantibodies against insulin (IAA), insulinoma-associated antigen-2 (IA-2), glutamic acid decarboxylase (GAD), zinc-transporter 8 (ZnT8) and islet cell antibodies (ICA) [195,196,197]. These autoantibodies may appear at any age, but the peak of the first islet autoantibody is usually before the age of 3 years [195,196,197,198]. Though the vast majority of autoantibodies related to T1DM recognize peptide antigens, antibodies against lipid antigens have also been described [17]. It has been shown that around 60% of sera from children with T1DM react against antigens composed of lysophospholipids [199], with many epitopes directed against gangliosides and sulfatide.

Gangliosides are sialic acid containing glycolipids, which are formed of Cer and an oligosaccharide chain. They are associated with the plasma membrane and in some cell types, including pancreatic islet cells are also present in the cytosol membranes like those of secretory granules. They play an important role in cell–cell interactions. Gangliosides are targets of a variety of anti-islet autoantibodies (please refer to the excellent review [200]). Early studies in the STZ-mouse model of autoimmune diabetes revealed that administration of a mixture of gangliosides (Cronassial, 150 mg/kg body wt., 21% GM1, 40% GD1a, 16% GD1b, 19% GT1b and 5% others) can dampen islet inflammation, but is not able to stop beta-cell destruction [201,202]. Dotta and colleagues were the first to identify GM2-1 in islets that was a target for IgG islet cell autoantibodies [203]. The content of GM2-1 was significantly overexpressed in NOD mice and the antibodies against this ganglioside were found to strongly correlate with T1DM progression in relatives of T1DM individuals [203,204]. Moreover GM2-1 was found to co-localize with insulin granule, similar to other autoantigens. GT3, GD3 and GM2-1 have been shown to be associated with severity of autoimmune reaction in T1DM [204]. Finally, in a case-control analysis of the Croatian population an association of B4GALNT1 gene variations (an enzyme involved in the biosynthesis of GM2 and GD2) with T1DM was detected [205], a phenomenon confirmed by Holm and colleagues in a larger cohort study of T1DM individuals [14].

Interestingly, recent studies in brain cancer have shown that an ER ATP-dependent chaperone GRP94 regulates the ratio of GM2-GM3 gangliosides [206]. The GM2-activator protein, which is a cofactor of beta-hexosaminidase responsible for GM2 hydrolysis to GM3, was shown to be a client for GRP94. Recent studies from the Marzec group have described a reduced expression of GRP94 in beta-cells from T2DM individuals [207] and its role in the inducible proteasome activation-mediated proinsulin degradation [208]. The GRP94 deficiency could disable the proper activity of the GM2-activator protein and thus prevent GM2 hydrolysis to GM3, linking cytokine effects with increased GM2 expression and islet autoimmunity in T1DM.

Moreover, GM1 and GD1a gangliosides have been shown to modulate inflammatory effects of LPS [209] by prevention of TLR4 translocation into lipid rafts. Interestingly, the activation of TLR4 and TLR3 was shown to be activated in viral infections in islets of T1DM patients, as well as in animal models of autoimmune diabetes and beta-cell lines [210,211,212,213]. Thus, the ganglioside pattern in beta-cells may be of crucial importance for beta-cell susceptibility to viral infections that are considered as one of major triggers of T1DM development [31,36,210,211,212,213,214,215,216,217,218]. The generation of tools for influencing ganglioside pattern in islets may represent an interesting new possibility to protect beta-cells from cytokine- and inflammation-mediated toxicity.

Concerning the role of sulfatide in T1DM development, this glycoSL and its precursor GalCer were shown to be ligands for CD14, the macrophage scavenger receptor, in a subset of beta-cells [219]. Anti-sulfatide antibodies have been detected in prediabetic and newly diagnosed T1DM patients [220,221] (excellently reviewed by Buschard [104]). Anti-sulfatide antibodies were shown to reduce insulin secretion and exocytosis from beta-cells [143].

Finally, sphingomyelin patches on plasma membrane act as epitopes for IC2, a monoclonal antibody that specifically recognizes the surface of beta-cells [222]. This raises the possibility that cell surface sphingomyelin pattern might be involved in the autoimmune reaction directed against beta-cells.

## 10. Conclusions and Perspectives

**Figure 3:**
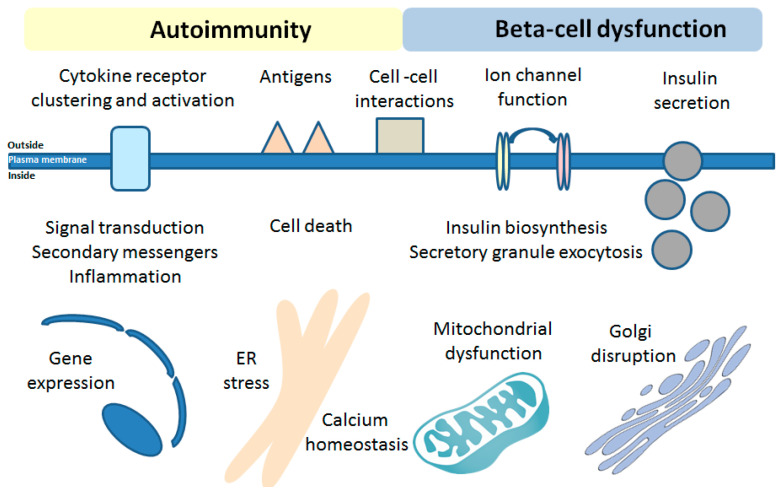
The possible involvement of sphingolipids in beta-cell biology during T1DM development. Rearrangements of SLs in response to the action of proinflammatory cytokines that are released by activated immune cells likely participate in islet autoimmunity, beta-cell dysfunction and death by multiple mechanisms.

The unique beta-cell sphingolipid rheostat and limitations of self-regulation upon T1DM development might serve as one of the major underlying mechanisms involved in beta-cell dysfunction and death in T1DM, similarly to neurodegenerative disorders. Further studies, involving the modern techniques to track the SL flow and de novo SL biosynthesis (such as [223,224]) as well as SL analogues and inhibitors of the SL pathway enzymes, should enable identification of novel SL-related pathways and targets that are engaged in human beta-cell susceptibility to proinflammatory cytokines during T1DM development. The availability of the excellent model human beta-cell line, EndoC-βH1 beta-cells [225,226], for in vitro studies will drive the progress of studies on the role of SLs in T1DM in upcoming years. The impact of beta-cell S1P homeostasis and SPL needs further investigations in beta-cell specific SL enzyme KO models. Based on so-far obtained data/observations, it is expected that SLs will be likely shown to affect all major functions of beta-cells and will provide crucial insights into the activation of islet autoimmunity (illustrated in Figure 3).

Certainly, upcoming years will bring the full characterization of the effects of cytokines on SLs of beta-cells leading to exciting new insights into mechanisms underlying cytokine-mediated beta-cell death, which shall show us how to help the vulnerable beta-cell facing T1DM.
